# A mixed-methods pilot study of handheld fan for breathlessness in interstitial lung disease

**DOI:** 10.1038/s41598-021-86326-8

**Published:** 2021-03-25

**Authors:** Yet H. Khor, Kirushallini Saravanan, Anne E. Holland, Joanna Y. T. Lee, Christopher J. Ryerson, Christine F. McDonald, Nicole S. L. Goh

**Affiliations:** 1 Department of Respiratory and Sleep Medicine, Austin Health, 145 Studley Road, Heidelberg, VIC 3084 Australia; 2 Institute for Breathing and Sleep, Heidelberg, VIC Australia; 3 Faculty of Medicine, University of Melbourne, Melbourne, VIC Australia; 4 Department of Respiratory Medicine, Alfred Health, Melbourne, Australia; 5 Department of Allergy, Immunology and Respiratory Medicine, Monash University, Melbourne, Australia; 6 Department of Physiotherapy, Alfred Health, Melbourne, Australia; 7 Centre for Heart Lung Innovation, Providence Health Care, Vancouver, BC Canada; 8 Department of Medicine, University of British Columbia, Vancouver, BC Canada

**Keywords:** Medical research, Signs and symptoms

## Abstract

Dyspnoea is a cardinal symptom of fibrotic interstitial lung disease (ILD), with a lack of proven effective therapies. With emerging evidence of the role of facial and nasal airflow for relieving breathlessness, this pilot study was conducted to examine the feasibility of conducting a clinical trial of a handheld fan (HHF) for dyspnoea management in patients with fibrotic ILD. In this mixed-methods, randomised, assessor-blinded, controlled trial, 30 participants with fibrotic ILD who were dyspnoeic with a modified Medical Research Council Dyspnoea grade ≥ 2 were randomised to a HHF for symptom control or no intervention for 2 weeks. Primary outcomes were trial feasibility, change in Dyspnoea-12 scores at Week 2, and participants’ perspectives on using a HHF for dyspnoea management. Study recruitment was completed within nine months at a single site. Successful assessor blinding was achieved in the fan group [Bang’s Blinding Index − 0.08 (95% CI − 0.45, 0.30)] but not the control group [0.47 (0.12, 0.81)]. There were no significant between-group differences for the change in Dyspnoea-12 or secondary efficacy outcomes. During qualitative interviews, participants reported that using the HHF relieved breathlessness and provided relaxation, despite initial scepticism about its therapeutic benefit. Oxygen-experienced participants described the HHF being easier to use, but not as effective for symptomatic relief, compared to oxygen therapy. Our results confirmed the feasibility of a clinical trial of a HHF in fibrotic ILD. There was a high level of patient acceptance of a HHF for managing dyspnoea, with patients reporting both symptomatic benefits and ease of use.

## Introduction

Patients with fibrotic interstitial lung disease (ILD) experience distressing breathlessness and reduced exercise tolerance that significantly impact quality of life. At presentation, 44–83% of patients with fibrotic ILD walk slower than people of similar age or need to stop when walking at their own pace on the level due to dyspnoea^1,2^. Despite recent advances in anti-fibrotic therapies that slow progression of fibrotic ILD^3–7^, these drugs are not effective in relieving symptoms or in improving health-related quality of life (HRQoL).

There is emerging evidence that facial and nasal airflow can reduce the sensation of breathlessness^8–10^. Handheld fans (HHFs) are inexpensive, portable, and readily available. More importantly, the use of a HHF may enhance self-efficacy for symptom management. In patients with dyspnoea secondary to other conditions, including chronic obstructive pulmonary disease (COPD) and malignancy, symptomatic benefits have been reported with the use of a HHF^11–13^.

The effects of a HHF for dyspnoea management in patients with fibrotic ILD have not been explored. This pilot study aimed to explore the feasibility and acceptability of a randomised controlled trial evaluating the use of a HHF for dyspnoea management in patients with fibrotic ILD.

## Methods

### Study design and participants

This mixed-methods, randomised, single-blinded, controlled trial was conducted at a quaternary hospital in Melbourne, Australia. Eligible participants were aged ≥ 18 years with fibrotic ILD who were dyspnoeic with a modified Medical Research Council (mMRC) Dyspnoea grade ≥ 2 and were not currently using a HHF. Fibrotic ILD was defined as a multidisciplinary diagnosis of chronic ILD of any aetiology^14^, with features of diffuse fibrosing lung disease of > 10% extent on high-resolution CT chest. Exclusion criteria included significant concurrent COPD (defined as FEV1/FVC < 60% on the most recent lung spirometry or extent of emphysema greater than extent of fibrosis on the most recent CT chest), and hospitalisation within the 4 weeks before screening. The study was approved by the Austin Health Research Ethics Board (HREC/46012/Austin-2018) and conducted according to the National Health and Medical Research Council’s National Statement on Ethical Conduct In Human Research (2007), and all subsequent updates, and in accordance with the Note for Guidance on Good Clinical Practice (CPMP/ICH/135/95), the Health Privacy Principles described in the Health Records Act 2001 (Vic) and Section 95A of the Privacy Act 1988 (and subsequent Guidelines). Individual informed consents were obtained for all participants. This study was registered with the Australian New Zealand Clinical Trials Registry (ACTRN12618001949279, date of trial registration: 30/11/2018).

### Interventions, randomisation and blinding

Thirty participants were randomised in a 1:1 ratio to either the intervention group (HHF for symptom control according to standardised instructions (Supplementary file 1: Standardised instructions for intervention) or the control group (no intervention) for 2 weeks, using a computer-generated sequence. Allocation concealment was achieved using sequentially numbered, opaque, sealed envelopes and was performed by an investigator without patient contact (A.E.H.). Given the lack of a plausible placebo, participants and clinicians were unblinded to the group assignments. Single blinding was achieved by ensuring the trial assessor (K.S.) was unaware of the allocation of study groups until the completion of study evaluation. This was accomplished by having a separate trial visit with an investigator (Y.H.K.) for intervention allocation and education, between 7 and 10 days from baseline assessment, in the absence of the trial assessor. The success of assessor blinding was measured at Week 2 using the Bang’s Blinding Index, with the assessor being asked to guess participants’ intervention allocation after the completion of trial assessment. Using the responses, the index is scaled to a continuum of − 1 to 1, with 1 being complete lack of blinding, 0 being perfect blinding, and − 1 being opposite guessing^15^.

### Study assessments

Assessments were performed by the blinded assessor at baseline (after eligibility screening) and at Week 2. At screening, the mMRC was used to assess potential participants’ dyspnoea-related functional disability for trial eligibility. The Dyspnoea-12, a 12-item validated assessment tool with both physical and affective components^16^, was used to evaluate the severity of dyspnoea. Health-related quality of life was examined using the King’s Brief Interstitial Lung Disease Questionnaire, a validated 15-item ILD-specific questionnaire that covers three domains: breathlessness and activities, chest symptoms, and psychological symptoms^17^. The Self-efficacy for Managing Chronic Disease 6-item Scale^18^ was used to measure participants’ confidence in self-management. Functional performance was measured using validated questionnaires to quantify physical disability for activities of daily living (Manchester Respiratory Activities of Daily Living Questionnaire)^19^ and life-space mobility (UAB Study of Aging Life-Space Assessment)^20^, and objectively using a multisensor activity monitor, the SenseWear Armband (Bodymedia Inc., Pittsburgh, PA, USA).

Individual semi-structured interviews were conducted with all participants who completed the study to evaluate participants’ perspectives regarding use of a HHF for dyspnoea management and their trial experiences. An interview topic guide was devised from the investigators’ clinical experience as well as a comprehensive literature review (Supplementary file 2: Interview topic guide)^13,21^. HHF usage frequency and patterns were evaluated through self-report during semi-structured interviews. All interviews were recorded digitally, transcribed verbatim, and anonymised for analysis.

### Study outcomes

Primary outcomes were trial feasibility, change in Dyspnoea-12 scores between baseline and Week 2, and participants' perspectives regarding the use of a HHF for managing their symptoms. Evaluation of trial feasibility included study recruitment, participant withdrawal, data completeness for outcome measures, trial assessor blinding, and participants’ trial experience. Secondary outcomes were changes in exploratory efficacy measurements at Week 2, including HRQoL, self-efficacy, functional performance (activities of daily living and life-space mobility assessments, physical activity levels), and participants’ self-reported usage of the HHF.

### Sample size

It was considered that a sample of 30 would be sufficient to inform trial feasibility for a Phase III randomised controlled trial, as well as to qualitatively evaluate participants’ perceptions.

### Statistical analysis

#### Quantitative analysis

Statistical analyses were performed using Stata (v16 StataCorp, USA). Descriptive analyses were conducted to evaluate trial feasibility outcomes. Between-group comparisons were performed using linear mixed models with intervention groups and time, adjusted for potential confounders (including age, sex, and lung function measurements). For comparisons of enrolled and non-enrolled patients, t tests or Mann–Whitney tests were used for parametric and non-parametric data, respectively, with data distribution being evaluated using the Shapiro–Wilk normality test. Level of statistical significance was set at *p* < 0.05.

#### Qualitative analysis

Methodological principles of grounded theory underpinned qualitative analysis of the interview transcripts, which were conducted independently by two investigators (Y.H.K. and J.L.). The transcripts were initially analysed using open coding, where they were read line-by-line and fragmented into descriptive codes to represent the data^22,23^. Codes were then organized hierarchically to form themes and the original transcripts were searched to refine the relationship between themes and codes. The final themes were agreed through iterative discussion between the two investigators.

## Results

**Figure 1:**
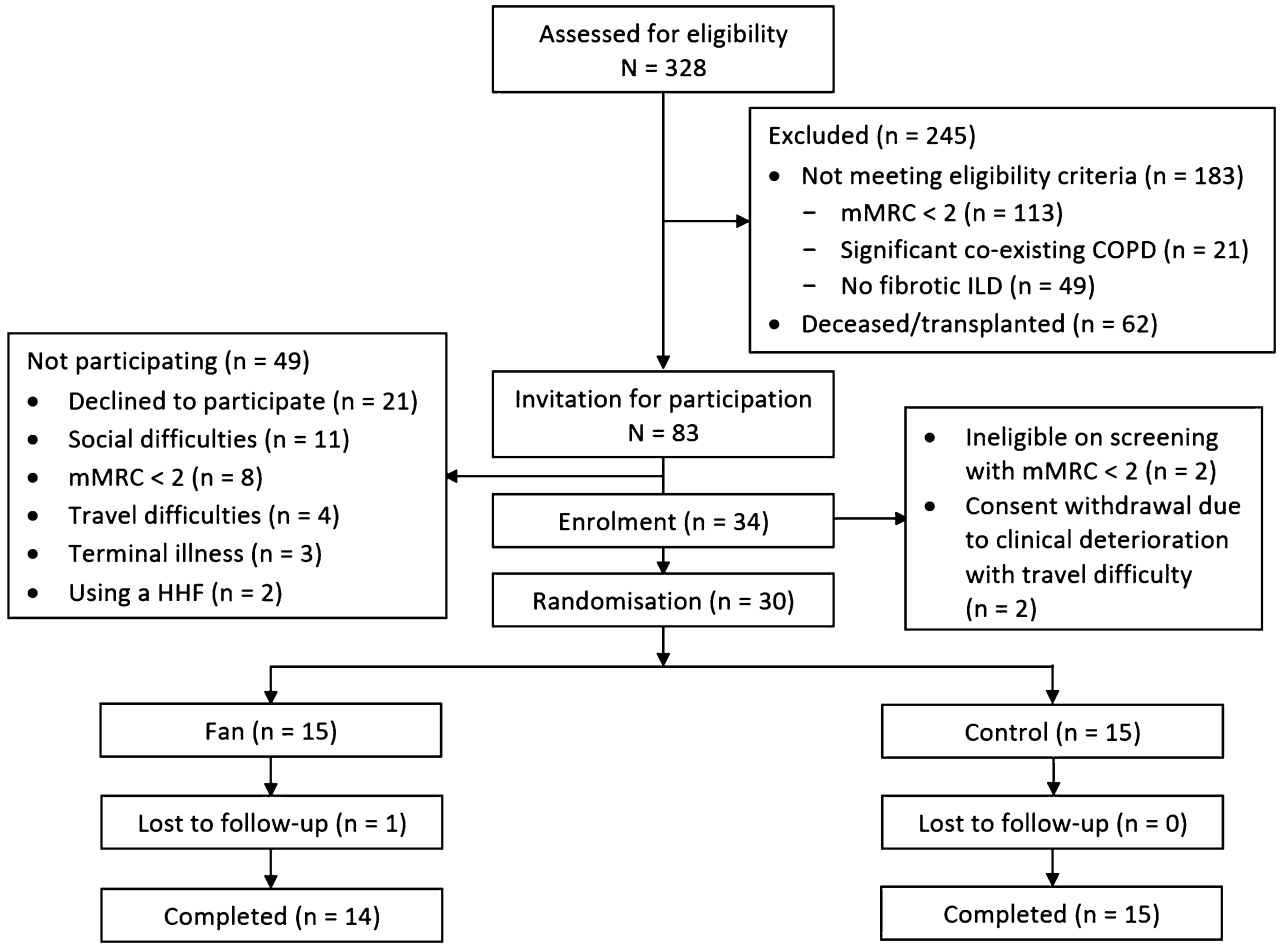
Study flow diagram.

Of 328 patients whose medical records were screened, 83 met the inclusion criteria and were invited for trial participation (Fig. 1). A total of 34 (41%) were enrolled in the study, with no significant differences in baseline characteristics, diagnoses, and lung function, compared to those who did not enrol (Supplementary file 3: Table E1). Of these, 30 were randomised with two not meeting the inclusion criterion of mMRC Dyspnoea grade ≥ 2 and two withdrawing their consent due to clinical deterioration requiring escalation of medical therapy. Baseline participant characteristics were similar in the two groups (Table 1). The most common diagnoses were idiopathic pulmonary fibrosis and connective tissue disease-related ILD. Seven participants were using domiciliary oxygen therapy at study enrolment. There were seven participants who were using a combination of inhaler therapies for management of asthma or mild COPD.

**Table 1:** Baseline participant characteristics.

	Intervention group (N = 15)	Control group (N = 15)
Age, years	73.7 ± 10.5	71.7 ± 7.3
Gender, female:male	8:7	6:9
BMI, kg/m^2^	28.9 ± 5.2	29.2 ± 5.3
Ever smoker, n (%)	9 (60)	10 (67)
Duration of diagnosis, years	3 (2, 8)	3 (2, 6)
**Diagnosis, n (%)**		
IPF	5 (33)	5 (33)
Non-IPF	10 (67)	10 (67)
Asbestosis	–	1
CTD-ILD	6	3
Drug-induced ILD	1	–
Fibrotic HP	1	3
Non-specific interstitial pneumonia	1	1
Unclassifiable ILD	1	2
FEV1/FVC	77.6 ± 6.0	79.3 ± 8.0
FEV1 (% pred)	78.2 ± 18.2	71.3 ± 15.0
FVC (% pred)	77.6 ± 18.0	68.2 ± 15.3
DLCO (% pred)	42.1 ± 11.5	41.7 ± 12.2
mMRC dyspnoea score	2 (2, 3)	2 (2, 3)
Domiciliary oxygen therapy, n (%)	2 (13)	5 (33)
**ILD therapies, n (%)**		
Anti-fibrotic therapy	4 (27)	4(27)
Immunosuppressant therapy	6 (40)	7 (47)
**Inhaler therapies, n (%)**		
Inhaled corticosteroid + long-acting beta-agonist	3 (20)	1 (7)
Long-acting muscarinic antagonist	1 (7)	1 (7)
Short-acting beta-agonist	5 (33)	2 (13)
Pulmonary rehabilitation in the previous 6 months, n (%)	7 (47)	8 (53)

Data are expressed as mean ± standard deviation or median (interquartile range), except where indicated.

*BMI* body mass index, *CTD-ILD* connective tissues disease-related interstitial lung disease, *DLCO* diffusing capacity for carbon monoxide, *FEV1* forced expiratory volume in one second, *FVC* forced vital capacity, *HP* hypersensitivity pneumonitis, *ILD* interstitial lung disease, *IPF* idiopathic pulmonary fibrosis, *mMRC* modified Medical Research Council.

### Primary outcomes

#### Trial feasibility

Study recruitment was completed within nine months, between February and November 2019, with an enrolment to randomisation ratio of 1.1:1. Only one participant (the fan group) withdrew from the study after randomisation due to social stresses with difficulty continuing study participation. Data completion rates were satisfactory, with 77% for SenseWear armband monitoring and 100% for all other outcome measures. Data incompleteness for SenseWear armband monitoring was due to insufficient device wearing time.

Evaluation of assessor blinding using the Bang’s blinding index revealed random guessing for the fan group [Index = −0.08 (95% confidence interval: − 0.45, 0.30)] and unsuccessful blinding for the control group [Index = 0.47 (95% confidence interval: 0.12, 0.81)].

All participants who completed the study reported positive experiences with this trial and stated they would recommend it to others if appropriate. Participants perceived minimal burden with their involvement in this trial. Most were motivated to advance research for symptom management in ILD and inspired to help others. At study completion, all participants, except for two in the control group, stated they would like to continue or start using a HHF.

#### Dyspnoea

There was no significant difference in the change of Dyspnoea-12 scores at Week 2 between groups [mean difference of − 2.2 (95% CI − 6.4, 1.9), *p* = 0.29; Table 2].

**Table 2:** Patient-reported outcome measures between baseline and Week 2.

	Fan group^a^		Control group^a^		Treatment effect^b^	*p* value
	Baseline	Week 2	Baseline	Week 2		
Dyspnoea-12	16.1 ± 2.2	13.4 ± 2.3	13.3 ± 2.2	12.8 ± 2.2	− 2.2 (− 6.4, 1.9)	0.29
K-BILD Breathlessness & Activities	29.2 ± 3.9	32.2 ± 3.9	32.5 ± 3.7	37.0 ± 3.7	− 1.5 (− 8.9, 5.9)	0.69
K-BILD Chest Symptoms	47.0 ± 5.2	56.1 ± 5.4	59.6 ± 5.0	58.8 ± 5.2	10 (− 5.1, 25.1)	0.19
K-BILD Psychological Symptoms	51.7 ± 4.2	53.4 ± 4.2	56.9 ± 4.1	57.5 ± 4.1	1.1 (− 4.1, 6.3)	0.40
K-BILD Total	48.0 ± 2.5	50.3 ± 2.5	52.3 ± 2.4	54.0 ± 2.4	0.7 (− 3.3, 4.7)	0.33
Self-efficacy^c^	5.4 ± 0.6	5.7 ± 0.7	5.5 ± 0.6	5.7 ± 0.6	0.07 (− 1.9, 2.0)	0.94
Activities of daily living^d^	14.4 ± 0.9	14.6 ± 1.0	15.1 ± 0.9	16.9 ± 0.9	− 2.5 (− 4.8, 0.3)	0.08
Life-space^e^	58.6 ± 6.3	58.0 ± 6.4	66.6 ± 6.1	63.7 ± 6.1	2.4 (− 10.4, 15.2)	0.72

Measured using: ^c^Self-efficacy for Managing Chronic Disease 6-item Scale; ^d^Manchester Respiratory Activities of Daily Living Questionnaire; ^e^University of Alabama at Birmingham Study of Aging Life-Space Assessment.

Data are expressed as ^a^mean ± standard error or ^b^mean difference (95% confidence interval). *P* values are between group comparison for mean difference.

*K-BILD* King’s Brief Interstitial Lung Disease.

#### Participants’ qualitative interviews

Three major and one minor theme emerged from the semi-structured interviews conducted with all completed participants (Table 3).

**Table 3:** Major and minor interview themes with illustrative quotes.

**Major themes**
Varying initial attitudes towards using a handheld fan as an intervention
I thought it was very unusual yeah, “I wonder how a fan would work,” so that’s what interested me the most. I couldn’t quite understand the concept of how a fan would work. (P8)
At the beginning, I was thinking, that’s a gimmick. (P27)
I thought about past experiences, riding the motorbike, keeping your face cool—you can breathe better. Or driving with the window down in the car. So would imagine having a fan would be beneficial. (P29)
Benefits of using a handheld fan
Just seemed to supply me with more air. Yeah, to get some air into my lungs. (P1)
I feel, I feel like uh, example, when you sweat and have cold drinks I feel better. When I have this one, I am like uh, hard with breathing, when I put this one, after I feel I breathe easy and uh, body is more comfortable. (P10)
It made me feel relaxed a bit more. (P17)
I think that the fan is something that is, secure. It’s something that you can put in your brain that is secure for you. It’s like a backup kind of thing. (P18)
I can use the fan in almost any place as long as it’s not noisy or intrusive. (P24)
Relative effects of handheld fans, oxygen, and inhaler therapies for symptom management
The fan helps a little bit more with it, you know, to help me breathe a bit better quicker (compared to inhaler therapies). Use my puffer a little bit less actually. (P2)
Not the same. The fan, it helps a little bit but, when I got really breathless, only the oxygen, oxygen helps more than the fan. I use the fan because it’s easier. (P4)
I believe it (the fan) helped more when I didn’t have the oxygen connected to me than when I did. Certainly it helped me in both cases, whether I had the oxygen or not. (P9)
**Minor theme**
Challenges of using a handheld fan
I’ve been to a lot of shops and, just can’t find any of these. All they have are those manual ones, that needs pumping. (P1)
I didn’t want to be in the public using something like that. It’s just embarrassing. (P15)

#### Major themes

1. Varying initial attitudes towards using a HHF as an intervention: Participants expressed different responses (12 positive, 12 uncertain, and 5 negative), when they were first introduced to the concept of using a HHF for symptom management. Some reported their scepticism and uncertainties with regards to the potential effects of using a HHF, while others anticipated beneficial effects based on their personal experiences, including airflow from overhead fans or riding in a vehicle.
2. Benefits of using a HHF: Most participants described symptomatic relief with using a HHF. In addition to providing facial airflow to relieve the sensation of dyspnoea, some participants expressed a sense of security and relaxation with using a HHF. They reported the ease of integrating the use of a HHF into their daily life.
3. Relative effects of HHF, oxygen, and inhaler therapies for dyspnoea management: Participants reported differential effects on breathlessness of HHF and existing interventions of oxygen and inhaler therapies. Oxygen-experienced participants described oxygen therapy provided greater relieving effects on breathlessness than the HHF, with potential additive effects when both were used together. Some participants favoured use of the HHF over inhaler therapies for symptom control, as the HHF resulted in a timelier improvement.

#### Minor theme

Challenges of using a HHF: Despite most participants describing the ease of using a HHF, a minority reported feeling embarrassed with using a HHF in public. Difficulties with sourcing a HHF were also raised by some participants who had previously attempted to get one.

### Secondary outcomes

#### Exploratory efficacy measurements

There were no significant differences between groups in HRQoL, self-efficacy, physical difficulties with activities of daily living, life-space mobility, or physical activity levels measured by SenseWear Armbands at Week 2 (Tables 2, 4).

**Table 4:** Physical activity levels measured using SenseWear armbands between baseline and Week 2.

	Fan group^a^		Control group^a^		Treatment effect^b^	*p* value
	Baseline	Week 2	Baseline	Week 2		
Steps per day	3423 ± 540	3620 ± 527	3082 ± 453	3206 ± 488	74 (− 807, 956)	0.87
Total energy expenditure (kCal/day)	8269 ± 343	8383 ± 340	8470 ± 288	8481 ± 287	104 (− 197, 404)	0.50
Total METs	1.10 ± 0.04	1.12 ± 0.04	1.05 ± 0.03	1.06 ± 0.03	0.003 (− 0.04, 0.44)	0.88
Duration of sedentary time per day (mins)	1129 ± 38	1139 ± 36	1205 ± 32	1149 ± 31	66 (− 30, 162)	0.18
Duration of time ≥ 3 METs per day^a^ (mins)	221 ± 27	243 ± 27	187 ± 23	183 ± 23	26 (− 16.1, 68.3)	0.23

Data are expressed as ^a^mean ± standard error or ^b^mean difference (95% confidence interval). *p* values are between group comparison.

*METs* metabolic equivalents of task.

#### Handheld fan usage

Most participants (71%) used the HHF for recovery after activities. They used it both in the home environment and for outdoor activities. Nine participants (64%) used their HHF daily, with the rest using it either weekly or for a few days a week. There was wide variability in the self-reported HHF usage duration for each episode, ranging from less than one minute to 20 min.

## Discussion

This pilot study demonstrated that a randomised controlled trial of HHF for dyspnoea management in patients with fibrotic ILD is feasible, although adequate assessor blinding was not achieved. There were no significant differences in patient-reported outcomes between groups, likely due to the limited intervention duration and small sample size in this pilot study. At study completion, participants expressed their willingness to use a HHF for self-management, although some were sceptical of its value initially. Participants described the HHF as being a helpful intervention for dyspnoea management, which could be easily adapted into daily life.

There is currently no established intervention that effectively controls dyspnoea in patients with fibrotic ILD. There is limited efficacy of various pharmacological agents for relieving dyspnoea in patients with ILD and other conditions, including opioids, benzodiazepines, and sertraline^24–27^. Recent studies have shown that ambulatory oxygen may improve quality of life and symptoms in patients with ILD and exertional desaturation^28,29^, although its longer-term impacts remain unknown. Our qualitative data and successful study recruitment highlight that patients with ILD are willing to participate in research evaluating non-pharmacological symptom management strategies. This is the first study that evaluated the possibility of using a HHF as an intervention for dyspnoea management in fibrotic ILD. Given the lack of feasibility in participant blinding for a HHF, we incorporated assessor blinding in this study, which was only achieved in the fan group. This confirms the challenge of blinding of group allocation for a non-pharmacological intervention such as the HHF, since participants may accidentally disclose their intervention allocation during assessments. Nevertheless, our study design and selected outcome measures using questionnaires and physical activity monitors were acceptable to participants, with high levels of data completeness.

The exact mechanisms by which the HHF aids relief of breathlessness remain unclear. A HHF may exert its effects by providing cooling of the facial skin and stimulation of oral and nasal mucosal flow receptors, which are innervated by the second and third branches of the trigeminal nerve^8,30,31^. Preliminary functional neuroimaging data suggest that facial airflow can alter neural activities within the brain and modulate the central perception of breathlessness^32^. A recent systematic review showed improved breathlessness both at rest and during exertion with facial and nasal airflow delivered by medical air or HHF^33^. In this study, participants described such sensations when using a HHF, with the additional benefits of relaxation and sense of security. Similar effects were reported in previous qualitative studies of patients with malignancy and COPD that evaluated the effects of a HHF for dyspnoea management, either as a sole intervention or in combination with a multi-component chronic breathlessness intervention program^13,20^. Our findings extend previous results, with some participants reporting differential effects and preference for using a HHF in comparison to oxygen and inhaler therapies for symptom control. The HHF provides an alternative option for dyspnoea management. However, the optimal timing and usage duration of a HHF, and how or whether it should be co-administered with other therapies, are unknown.

Dyspnoea perception is a result of complex interactions of physiological, psychosocial, and social and environmental factors^34,35^. Thus, it would seem likely that optimal management of breathlessness in advanced lung disease would require a combination of pharmacological and non-pharmacological interventions^36^. However, in general, the utilisation of non-pharmacological interventions is lower than pharmacological agents in medical care, with patients’ acceptance being an important factor^37–39^. Given the simplicity of a HHF, many participants disbelieved its therapeutic potential at the outset of this study. This highlights the importance of providing adequate explanation regarding the purported scientific rationale of using a HHF with instructions and/or demonstration of how to use the HHF in the appropriate facial region, when prescribing one for managing breathlessness in clinical practice. Patients’ negative expectations of an intervention can have undesirable effects on treatment outcomes and adherence, the so-called nocebo effect^40^. An effective patient-clinician relationship may improve patients’ acceptance of such an intervention^41^.

This study has several limitations. In addition to inadequate blinding of assessors, there was a lack of an appropriate control intervention and participant blinding in this study. Both selecting a control intervention and blinding are crucial fundamental methodological components in clinical trials, in order to minimise differential management or assessment and prevent biased estimates of treatment effects. However, blinding of participants and appropriately matching the control intervention of a HHF would be very challenging. This study was not designed to assess intervention efficacy, but to evaluate the appropriateness of different outcome measures and patients’ acceptability of the intervention. Although participants reported benefits from using a HHF, its true efficacy and the magnitude of its effects, if any, are unclear. Anxiety and psychological well-being should be evaluated in future studies, given participants’ perception of increased sensation of security with using a HHF. There was wide variability in HHF usage patterns among participants, which could impact on the evaluation of its intervention effects. Given differences in participants’ lifestyles and home environments, the instructions for HHF usage suggested that participants should use the HHF according to their daily needs, but optimal strategies for using a HHF for dyspnoea management remains unknown. Nevertheless, a low HHF usage level may dampen its potential intervention effects. Usage of the HHF was not objectively measured, so it was not possible to evaluate any relationship between greater hours of use and perceived efficacy. Electronic measurement of usage could be considered for future trials. While there was a small proportion of participants with co-existing obstructive lung disease, we ensured that these participants had primarily fibrotic ILD by excluding those with significant obstructive ventilatory defects or predominant emphysematous changes on CT chest. The control group had a lower mean FVC and more participants on domiciliary oxygen therapy than the intervention group, which may have influenced the efficacy outcomes. Patients with different severities of dyspnoea and lung disease may have varying responses to therapeutic effects of the HHF. Previous positive studies of HHF had predominantly evaluated patients with severe COPD or advanced malignancy associated with high symptom burden^11,33^.

## Conclusion

This mixed-methods pilot study confirmed the feasibility of conducting a randomised controlled trial to evaluate the use of a HHF for dyspnoea management in patients with fibrotic ILD, although adequate trial assessor blinding was not achieved. This is the first study to suggest that a HHF can be used for dyspnoea management in fibrotic ILD, with adequate patient education needed at the intervention initiation to ensure patient understanding and acceptance of the device.

## Supplementary Information

Supplementary Informations.

## Data Availability

The datasets used and/or analysed during the current study are available from the corresponding author on reasonable request.
