# Supplementary material for: A mixed-methods pilot study of handheld fan for breathlessness in interstitial lung disease
Source: Sci Rep. 2021 Mar 25;11:6874. doi: 10.1038/s41598-021-86326-8 (PMC7994303; doi:10.1038/s41598-021-86326-8)
Supplement: Supplementary file 1 — Supplementary Informations. [file 41598_2021_86326_MOESM1_ESM.pdf]

Online Supplementary:

Manuscript Title:
A mixed-methods pilot study of handheld fan for breathlessness in interstitial lung disease

Authors:
Yet H Khor, Kirushallini Saravanan, Anne E Holland, Joanna YT Lee, Christopher J Ryerson, Christine F McDonald, Nicole SL Goh

Table of content:

- Supplementary file 1: Standardised instructions for intervention ………………………………… 3
- Supplementary file 2: Interview topic guide ………………………………………………………………. 4
- Supplementary file 3: Table E1 - Baseline participant characteristics of enrolled and non-enrolled patients ……………………………………………………………………………………………………….. 5

Participant Information – Handheld Fan

How should I use the handheld fan?

- Hold the fan approximately 15cm from your face.
- Aim the draft of air towards the central part of your face so that you can feel it around the sides of your nose and above your top lip.

When can I use it?

- You can use the fan as often as you like
- You may find it useful to use your fan:
    - during activities
    - when you need a break after activities
    - when you feel breathless at rest
- You can use your fan with other interventions, such as relaxation, breathing exercises and oxygen therapy.

Keep your fan in your pocket or bag when you go out
Keep your fan next to you when you are at home

Interview Topic Guide

<u>All participants</u>

1. How does your chest condition affect you at the moment? (probe: daily life? particular problems? exercise tolerance?)
2. How do you manage your symptoms? (probe: any treatment, such as drugs, oxygen)
3. If the participant is using other interventions for managing symptoms: How do you find the treatment? (probe: why is that?)
4. What do you think about this trial? (probe: why is that?)
5. What were your thoughts when you heard about using a handheld fan for managing symptoms? (probe: why is that?)

<u>For participants using a handheld fan</u>

6. How often did you use the handheld fan? (probe: daily, a few times a week, occasionally, not at all?)
7. Tell me about how you use the handheld fan. (probe: when? During activities, during recovery, at rest)
8. If the participant is using other interventions for managing symptoms: How do you compare using the handheld fan with your other treatments? (probe: why is that?)
9. What will stop you from using a handheld fan?
10. Do you want to keep using a handheld fan? (probe: why is that?)

<u>For participants not using a handheld fan</u>

11. Do you want to start using a handheld fan? (probe: why is that?)

<u>Conclusion</u>

12. Do you have anything else about this trial that you would like to share?

Table E1: Baseline participant characteristics of enrolled and non-enrolled patients

	**Enrolled Group (N = 34)**	**Non-Enrolled Group (N = 49)**	**p-value**
**Age, years**	73.3 ± 8.6	71.2 ± 14.7	0.47
**Gender, Female:Male**	16:18	25:24	0.82
**BMI, kg/m^2**	28.8 ± 5.0	29.4 ± 5.2	0.78
**Ever smoker, n (%)**	20 (59)	24 (49)	0.50
**Duration of diagnosis, years**	3 (2, 6)	3 (1.5, 6)	0.69
**Diagnosis, n (%)**			1.00
• **IPF**	11 (32)	16 (33)	
• **Non-IPF**	23 (68)	33 (67)	
**FEV1/FVC**	78.8 ± 6.8	79.4 ± 6.4	0.56
**FVC (% pred)**	70.7 ± 17.5	74.8 ± 17.8	0.62
**DLCO (% pred)**	42.1 ± 11.5	46.9 ± 17.3	0.19

Data are expressed as mean ± standard deviation or median (interquartile range), except where indicated.

Abbreviations: BMI, body mass index; DLCO, diffusing capacity for carbon monoxide; FER, forced expiratory ratio; FEV1, forced expiratory volume in one second; FVC, forced vital capacity; IPF, idiopathic pulmonary fibrosis
